# Supplementary material for: Assessing the quantitative relationships between the impervious surface area and surface heat island effect during urban expansion
Source: PeerJ. 2021 Jul 23;9:e11854. doi: 10.7717/peerj.11854 (PMC8312497; doi:10.7717/peerj.11854)
Supplement: Supplemental Information 1 [file peerj-09-11854-s001.zip › Dataset/Dataset.docx]

All the data in this article comes from remote sensing data. For example: Landsat satellite data (including ETM+, TM and Operational Land Imager (OLI) Thermal Infrared Scanner (TIRS) data) are downloaded from the United States Geological Survey (USGS) website (<https://glovis.usgs.gov/>). See Table 1 for specific data information.

**Table 1.** Data descriptions

| **Sensor** | **Date** | **GTM** | **Scene ID** | **Spatial resolution(m)** |
| --- | --- | --- | --- | --- |
| ETM+ | 30/4/2002 | 03:23:48 | LE71290432002120SGS00 | 30 |
| TM | 6/4/2008 | 03:24:30 | LT51290432008097BKT00 | 30 |
| OLI | 23/4/2014 | 03:34:51 | LC81290432014113LGN02 | 30 |
| OLI | 9/5/2020 | 03:34:22 | LC81290432020130LGN00 | 30 |
